# Deep-learning-driven optical coherence tomography analysis for cardiovascular outcome prediction in patients with acute coronary syndrome

**DOI:** 10.1093/ehjdh/ztae067

**Published:** 2024-09-27

**Authors:** Tomoyo Hamana, Makoto Nishimori, Satoki Shibata, Hiroyuki Kawamori, Takayoshi Toba, Takashi Hiromasa, Shunsuke Kakizaki, Satoru Sasaki, Hiroyuki Fujii, Yuto Osumi, Seigo Iwane, Tetsuya Yamamoto, Shota Naniwa, Yuki Sakamoto, Yuta Fukuishi, Koshi Matsuhama, Hiroshi Tsunamoto, Hiroya Okamoto, Kotaro Higuchi, Tatsuya Kitagawa, Masakazu Shinohara, Koji Kuroda, Masamichi Iwasaki, Amane Kozuki, Junya Shite, Tomofumi Takaya, Ken-ichi Hirata, Hiromasa Otake

**Affiliations:** Division of Cardiovascular Medicine, Department of Internal Medicine, Kobe University Graduate School of Medicine, Kobe, Japan; Division of Cardiovascular Medicine, Department of Internal Medicine, Kobe University Graduate School of Medicine, Kobe, Japan; Division of Molecular Epidemiology, Kobe University Graduate School of Medicine, Kobe, Japan; Division of Molecular Epidemiology, Kobe University Graduate School of Medicine, Kobe, Japan; Division of Cardiovascular Medicine, Department of Internal Medicine, Kobe University Graduate School of Medicine, Kobe, Japan; Division of Cardiovascular Medicine, Department of Internal Medicine, Kobe University Graduate School of Medicine, Kobe, Japan; Division of Cardiovascular Medicine, Department of Internal Medicine, Kobe University Graduate School of Medicine, Kobe, Japan; Division of Cardiovascular Medicine, Department of Internal Medicine, Kobe University Graduate School of Medicine, Kobe, Japan; Division of Cardiovascular Medicine, Osaka Saiseikai Nakatsu Hospital, Osaka, Japan; Division of Cardiovascular Medicine, Department of Internal Medicine, Kobe University Graduate School of Medicine, Kobe, Japan; Division of Cardiovascular Medicine, Department of Internal Medicine, Kobe University Graduate School of Medicine, Kobe, Japan; Division of Cardiovascular Medicine, Department of Internal Medicine, Kobe University Graduate School of Medicine, Kobe, Japan; Division of Cardiovascular Medicine, Department of Internal Medicine, Kobe University Graduate School of Medicine, Kobe, Japan; Division of Cardiovascular Medicine, Department of Internal Medicine, Kobe University Graduate School of Medicine, Kobe, Japan; Division of Cardiovascular Medicine, Department of Internal Medicine, Kobe University Graduate School of Medicine, Kobe, Japan; Division of Cardiovascular Medicine, Department of Internal Medicine, Kobe University Graduate School of Medicine, Kobe, Japan; Division of Cardiovascular Medicine, Department of Internal Medicine, Kobe University Graduate School of Medicine, Kobe, Japan; Division of Cardiovascular Medicine, Department of Internal Medicine, Kobe University Graduate School of Medicine, Kobe, Japan; Division of Cardiovascular Medicine, Department of Internal Medicine, Kobe University Graduate School of Medicine, Kobe, Japan; Division of Cardiovascular Medicine, Department of Internal Medicine, Kobe University Graduate School of Medicine, Kobe, Japan; Division of Cardiovascular Medicine, Department of Internal Medicine, Kobe University Graduate School of Medicine, Kobe, Japan; Division of Cardiovascular Medicine, Department of Internal Medicine, Kobe University Graduate School of Medicine, Kobe, Japan; Division of Molecular Epidemiology, Kobe University Graduate School of Medicine, Kobe, Japan; Department of Cardiology, Hyogo Prefectural Awaji Medical Centre, Sumoto, Japan; Department of Cardiology, Hyogo Prefectural Awaji Medical Centre, Sumoto, Japan; Division of Cardiovascular Medicine, Osaka Saiseikai Nakatsu Hospital, Osaka, Japan; Division of Cardiovascular Medicine, Osaka Saiseikai Nakatsu Hospital, Osaka, Japan; Division of Cardiovascular Medicine, Hyogo Prefectural Harima-Himeji General Medical Centre, Himeji, Japan; Division of Cardiovascular Medicine, Department of Internal Medicine, Kobe University Graduate School of Medicine, Kobe, Japan; Division of Cardiovascular Medicine, Department of Internal Medicine, Kobe University Graduate School of Medicine, Kobe, Japan

**Keywords:** Optical coherence tomography, Deep learning, Survival model, Acute coronary syndrome

## Abstract

**Aims:**

Optical coherence tomography (OCT) can identify high-risk plaques indicative of worsening prognosis in patients with acute coronary syndrome (ACS). However, manual OCT analysis has several limitations. In this study, we aim to construct a deep-learning model capable of automatically predicting ACS prognosis from patient OCT images following percutaneous coronary intervention (PCI).

**Methods and results:**

Post-PCI OCT images from 418 patients with ACS were input into a deep-learning model comprising a convolutional neural network (CNN) and transformer. The primary endpoint was target vessel failure (TVF). Model performances were evaluated using Harrell’s *C*-index and compared against conventional models based on human observation of quantitative (minimum lumen area, minimum stent area, average reference lumen area, stent expansion ratio, and lesion length) and qualitative (irregular protrusion, stent thrombus, malapposition, major stent edge dissection, and thin-cap fibroatheroma) factors. GradCAM activation maps were created after extracting attention layers by using the transformer architecture. A total of 60 patients experienced TVF during follow-up (median 961 days). The *C*-index for predicting TVF was 0.796 in the deep-learning model, which was significantly higher than that of the conventional model comprising only quantitative factors (*C*-index: 0.640) and comparable to that of the conventional model, including both quantitative and qualitative factors (*C*-index: 0.789). GradCAM heat maps revealed high activation corresponding to well-known high-risk OCT features.

**Conclusion:**

The CNN and transformer-based deep-learning model enabled fully automatic prognostic prediction in patients with ACS, with a predictive ability comparable to a conventional survival model using manual human analysis.

**Clinical Trial Registration:**

The study was registered in the University Hospital Medical Information Network Clinical Trial Registry (UMIN000049237).

## Graphical Abstract

**Graphical Abstract:**
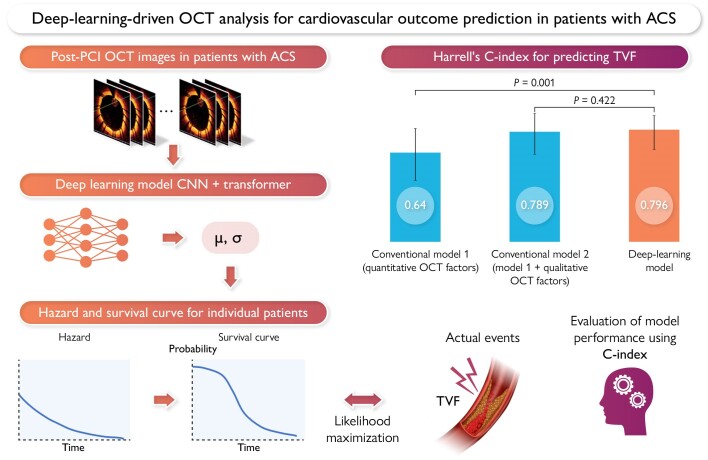
Deep-learning-driven optical coherence tomography (OCT) analysis for cardiovascular outcome prediction in patients with acute coronary syndrome (ACS). A deep-learning model utilizing a convolutional neural network (CNN) and a transformer enables automatic prediction of cardiovascular outcomes from post-percutaneous coronary intervention (PCI) OCT images in patients with ACS. Harrell’s *C*-index is significantly higher than that of the conventional model including only quantitative factors, and comparable to that of the conventional model including both quantitative and qualitative factors.

## Introduction

Despite advancements in interventional techniques and optimal medical therapies, acute coronary syndrome (ACS) continues to be a leading cause of morbidity and mortality worldwide. Given that ACS is typically accompanied by systemic atherosclerotic complications, affected patients have a high incidence of cardiovascular events, even after successful percutaneous coronary intervention (PCI).^1^ Previous studies have clarified several critical intravascular findings after PCI to identify high-risk intracoronary conditions that can lead to future cardiovascular events.^1,2^

Optical coherence tomography (OCT) is an intravascular imaging modality that allows the detailed evaluation of intracoronary microstructures post PCI.^3^ Previous studies have demonstrated that OCT-guided stent optimization based on quantitative OCT evaluation is associated with better clinical outcomes in patients with ACS.^4^ Additionally, qualitative OCT assessment and identification of features such as lipid-rich plaque and thin-cap fibroatheroma (TCFA) in non-culprit lesions (NCLs) have been associated with subsequent adverse clinical events after PCI.^5–7^ However, conventional human-based OCT analysis has inherent limitations, including the need for specialized knowledge and experience, time, and effort, objectivity and reproducibility issues, and limited feature extraction by human observers.^8^

Recently, deep-learning technology has made remarkable progress and has already been implemented in the medical field. This technique has also been successfully applied in the field of cardiovascular OCT for plaque classification, segmentation, and image reconstruction.^8–11^ A previous study reported that artificial intelligence–based software can detect vulnerable plaques that are potentially associated with poor prognosis.^12^ However, the mere classification of vulnerable plaques alone may not be sufficient for predicting poor outcomes, as most plaques, identified through a single-time-point assessment, heal naturally and do not correlate consistently with clinical events. Therefore, in this study we aim to establish an artificial intelligence–based survival model that predicts the prognosis directly from OCT images after PCI in patients with ACS.

## Methods

### Study design

This multicentre, retrospective, observational study used the Kobe University ACS-OCT registry to explore the relationship between the morphological plaque characteristics of culprit lesions in ACS and clinical outcomes. Consecutive patients with ACS who underwent OCT-guided PCI at four institutions between November 2014 and December 2020 were included in this study. The inclusion criteria were as follows: patients with *de novo* ACS who (i) underwent OCT-guided PCI with a drug-eluting stent and (ii) were aged ≥20 years. The participating institutions and exclusion criteria are described in Supplementary material online, *Appendices S1* and *S2*, respectively. The study protocol complied with the Declaration of Helsinki and was approved by the Ethics Committee of Kobe University Hospital. Informed consent was obtained in the form of an opt-out on the website of the Division of Cardiovascular Medicine at Kobe University Graduate School of Medicine. The study was registered in the University Hospital Medical Information Network Clinical Trial Registry (UMIN000049237).

### Outcomes

The primary endpoint was target vessel failure (TVF), including cardiac death, target vessel–related myocardial infarction (MI), and ischaemia-driven target vessel revascularization (TVR).^13^ Detailed definitions of outcomes are described in the Supplementary material online, *Appendix S3*. Clinical outcomes were determined by reviewing medical records and confirmed through direct contact with the patients, their families, or physicians.

### Conventional optical coherence tomography image analysis and definitions

At the end of the PCI procedure, OCT images were acquired using frequency-domain OCT (ILUMIEN; Abbott Vascular, Santa Clara, CA, USA) with a Dragonfly Optis OCT imaging catheter (Abbott Vascular). Offline OCT analysis was performed using dedicated software (Light Lab Imaging Inc., Westford, MA, USA) by independent observers blinded to the clinical presentation and lesion characteristics of the patient. The longitudinal image length was 75 mm at a pullback speed of 18 mm/s or 54 mm at a pullback speed of 36 mm/s.

The target vessel was divided into the following longitudinal sub-segments: (i) stented segments, (ii) adjacent reference segments (≤5 mm long), and (iii) NCLs.^6^ An NCL was defined as an untreated coronary segment with >30% diameter stenosis on angiography, located at least 5 mm away from the stent. In cases where multiple candidate NCLs were present, the most stenotic lesion was defined as the NCL (see Supplementary material online, *Figure S1*). For quantitative analysis, cross-sectional OCT images were measured for every frame at 0.1 or 0.2 mm intervals. A qualitative assessment was performed to evaluate irregular protrusion, stent thrombus, stent malapposition, major stent edge dissection, and TCFA in the NCL. Detailed definitions are provided in Supplementary material online, *Appendices S4* and *S5*.

### Data pre-processing

The entire data set was divided into quintiles, each representing 20% of the entire data set. One quintile was allocated as the test data set, while the remaining four quintiles were used for cross-validation and subdivided into training and validation data sets in a 3:1 ratio. To account for the varying number of pullbacks from the various participating centres, all data were aggregated and then randomly divided into five equal parts to ensure a balanced and representative division of the data set. Data in the Digital Imaging and Communications in Medicine format were converted into matrix form and resized to a resolution of 250 × 250 pixels. For the OCT images, areas outside the scanning range were replaced with zeros, and any text, such as examination information, was masked.

The images, initially 8-bit in depth, were standardized by dividing their pixel values by 256. Detailed data augmentation procedures are described in Supplementary material online, *Appendix S7*.

Additionally, to further validate the model’s performance, we performed prospective validation at one of the participating facilities. Comprehensive details are provided in Supplementary material online, *Appendix S8*.

### Deep-learning model

**Figure 1:**
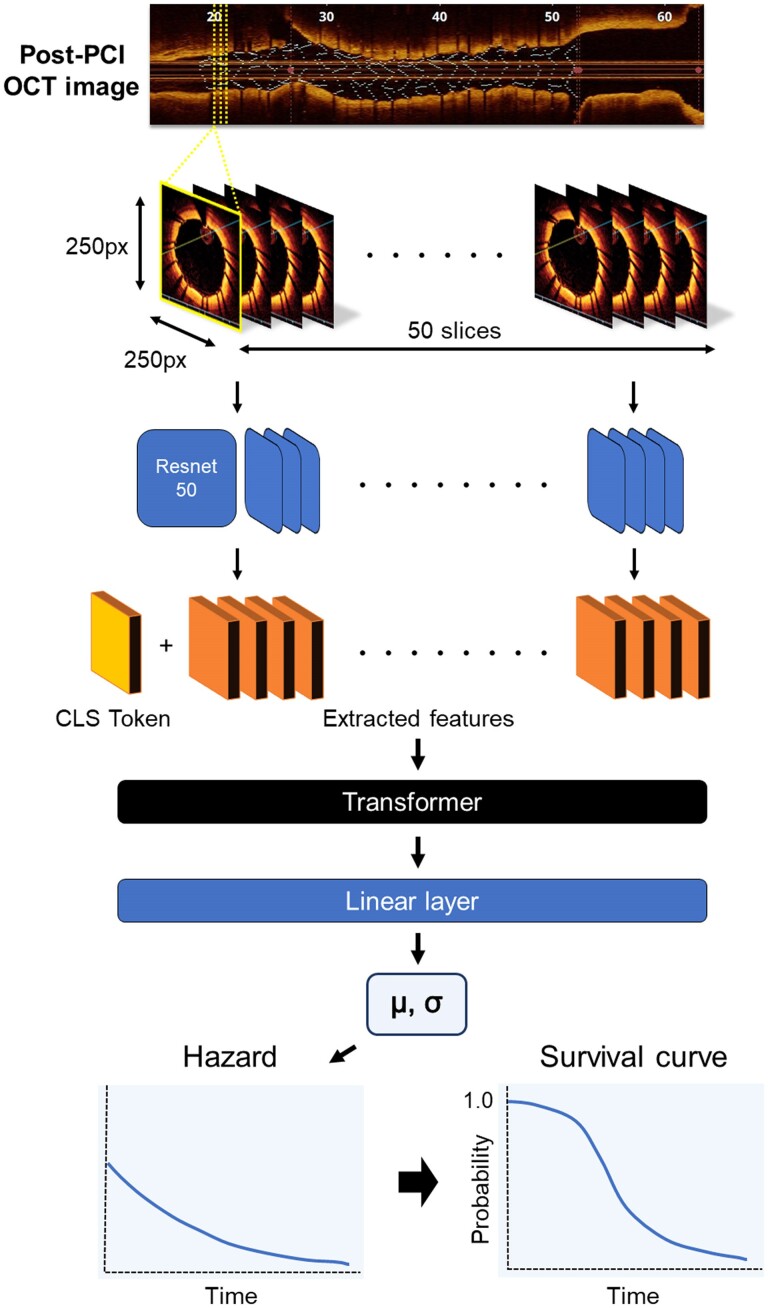
A schematic overview of the deep-learning model. Post-percutaneous coronary intervention optical coherence tomography images consisting of 50 random slices (250 × 250 pixels) are input into the convolutional neural network (Resnet 50), generating 256 element vectors. These vectors are fed into a transformer encoder model, along with a class token. Following layer normalization, a linear layer outputs two patient-specific parameters—*µ* and *σ*—that define the patient-specific hazard curve. The weights are optimized using the maximum likelihood process to match the actual survival data. Finally, the optimized hazard function is used to predict patient-specific survival curves.

We developed a hybrid model that integrated a convolutional neural network (CNN)^14^ with a transformer model.^15^ The feature extraction component of our model utilized Resnet50,^16^ a specific CNN architecture, pre-trained on the ImageNet data set. This model processes cross-sectional OCT images to generate feature vectors, each consisting of 256 elements. For each epoch, 50 slices were randomly selected from the entire length of the vessel, ensuring the model was exposed to a diverse representation of vessel segments. Given a set of 50 cross-sectional images, the feature matrix for each sample was dimensioned at (50, 256), and the CNN model employed common weights to embed each image into the same 256-dimensional feature space. Subsequently, the features extracted by the CNN, along with a class token of the same element number, were input into a transformer encoder model. The feature vector derived from the output class token was extracted, layer normalization applied, and then fed into a linear layer, outputting two values: *µ* and *σ* (*Figure 1*). These two parameters represent location and scale, as assumed by the time to the event with a log-logistic distribution. A negative log-likelihood loss was computed from the two values, *µ* and *σ*, using the following equation:


(1) $$\begin{array}{rl} \mathrm{nll} & =-\log\;L\\ & =\underset{i}{\sum }\;\left[\frac{\log\;x_{i}-\mu_{i}}{\sigma_{i}}+\delta_{i}\log\;\sigma_{i}+(1+\delta_{i})\log\;\left[1+\exp\;\left(-\frac{\log\;x_{i}-\mu_{i}}{\sigma_{i}}\right)\right]\right], \end{array}$$

where *x_i_* is the observed time and *δ_i_* is the presence or absence of an event.^17^

The transformer model parameters were configured with a batch size of six, a learning rate ranging from 1e−3 to 1e−7 (log uniform distribution), and the Adam optimizer. The optimal model was determined by parameter exploration using the training and validation data sets. For each of the four folds, the model that achieved the highest *C*-index on the validation data set was saved. The top four models were then used to construct an ensemble model. The predictions from these models were combined by averaging the time-dependent values of the output survival curves, resulting in the final ensemble model’s prediction.

### Model evaluations and statistics

The performance of the deep-learning model was assessed using Harrell’s concordance index (*C*-index). The evaluation metric was defined as the time at which the survival curves indicated a 50% probability of survival. The survival function, *S(t*), was delineated using values of *µ* and *σ* produced by the deep-learning model, as given by the following equation:


(2) $$\begin{array}{c} S(t)=\frac{1}{1+\exp\;\left(\frac{\left(\log\;t\;-\;\mu \right)}{\sigma }\right)} \end{array}$$

The *C*-index was used as the evaluation metric for the Cox regression model. Both the Cox regression and deep-learning models were fitted using the training and validation data sets and subsequently evaluated using the test data set. The 95% confidence interval (CI) for each model metric was calculated using the bootstrap method with 1000 iterations. Furthermore, the comparison of model performance was based on the evaluation of each model’s metrics using identical bootstrap samples, with a two-sided significance test conducted to calculate the *P*-value. The base program was developed using Python 3.8 with the PyTorch 1.8 framework as the deep-learning library. The model visualization methods are shown in Supplementary material online, *Appendix S9*.

## Results

### Study population

**Figure 2:**
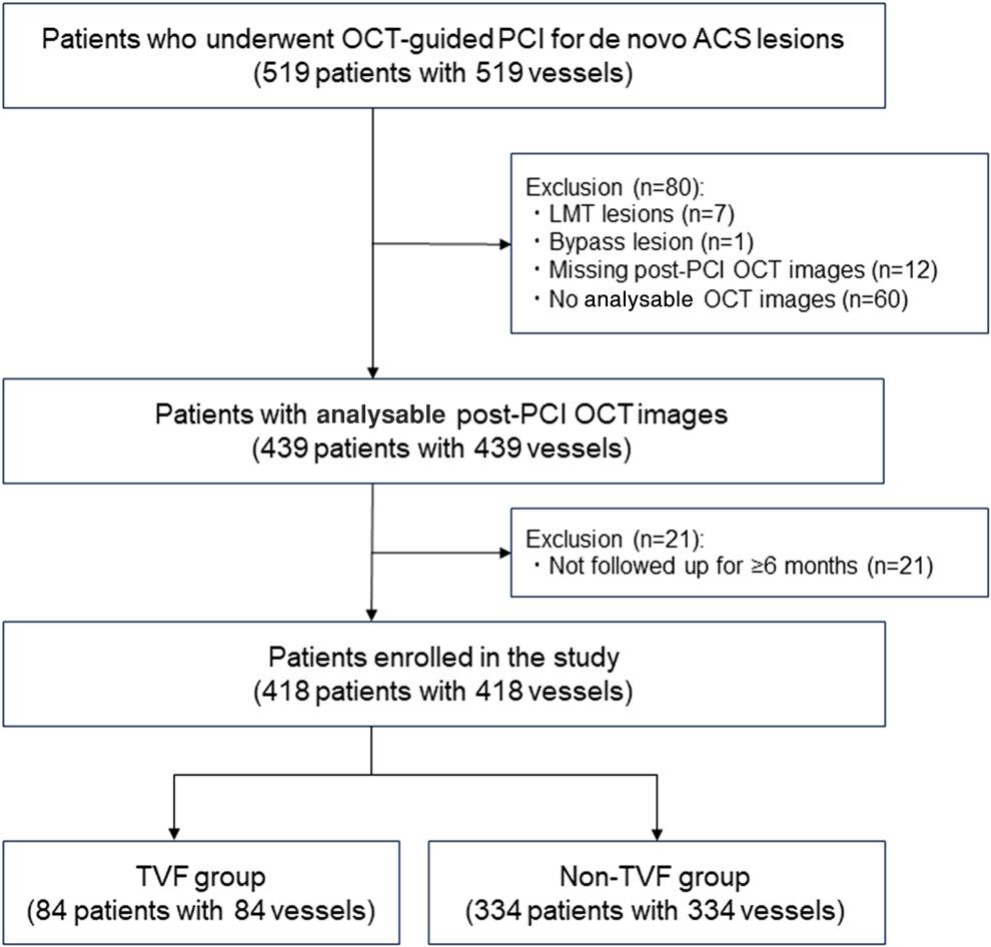
A patient flow chart.

In total, 519 consecutive patients underwent OCT-guided PCI for *de novo* ACS lesions during the study period. After excluding 101 patients, 418 patients with 418 vessels were included in the analysis. Among them, 359 patients overlapped with those from our previous study (*Figure 2*).^5^ Specifically, 225 NCLs were located in the proximal portion of the stent, while 193 NCLs were located in the distal portion. Among these patients, 334 (80%) were allocated to the training and validation data sets, with the remaining 84 (20%) assigned to the test data set.

The baseline patient, lesion, and conventional human-based OCT characteristics of each item in the data set are summarized in *Table 1*. The median age was 68 years, and 75.1% of patients were male. Patients presenting with ST-segment elevation MI accounted for 59.1% of the total study population. There were no significant differences in the baseline characteristics between the two data sets, except for serum creatinine levels. The reproducibility of the conventional OCT analysis is outlined in Supplementary material online, *Appendix S10* and *Figure S2*.

**Table 1:** Baseline patient and lesion characteristics and optical coherence tomography findings by human analysis

	Total (*n* = 418)	Training and validation (*n* = 334)	Test (*n* = 84)	*P*-value
Age, year	68 (60–76)	69 (60–77)	67 (60–75)	0.34
Male	314 (75.1)	253 (75.7)	61 (72.6)	0.55
BMI, kg/m^2^	23.3 (21.2–25.4)	23.2 (21.0–25.3)	23.5 (21.7–25.4)	0.29
Comorbidity				
Hypertension	278 (66.5)	216 (64.7)	62 (73.8)	0.11
Dyslipidaemia	259 (62.0)	203 (60.8)	56 (66.7)	0.32
Diabetes mellitus	168 (40.2)	131 (39.2)	37 (44.0)	0.42
Smoking	255 (61.0)	200 (59.9)	55 (65.5)	0.35
Family history	74 (17.7)	56 (16.8)	18 (21.4)	0.32
Haemodialysis	10 (2.4)	9 (2.7)	1 (1.2)	0.42
Prior MI	17 (4.1)	15 (4.5)	2 (2.4)	0.38
Prior PCI	25 (6.0)	21 (6.3)	4 (4.8)	0.60
Prior CABG	2 (0.5)	2 (0.6)	0 (0.0)	0.48
Clinical presentation				0.07
STEMI	247 (59.1)	193 (57.8)	54 (64.3)	
Non-STEMI	119 (28.5)	103 (30.8)	16 (19.0)	
Unstable angina	52 (12.4)	38 (11.4)	14 (16.7)	
Laboratory data				
LDL-C, mg/dL	126 (100–150)	124 (100–148)	131 (105–156)	0.13
HDL-C, mg/dL	46 (38–55)	46 (38–55)	45 (37–53)	0.40
TG, mg/dL	121 (77–192)	119 (76–192)	131 (84–196)	0.24
HbA1c, %	6.0 (5.7–6.7)	6.0 (5.6–6.6)	6.1 (5.7–7.5)	0.050
Creatinine, mg/dL	0.80 (0.68–0.94)	0.81 (0.69–0.94)	0.74 (0.65–0.89)	0.029
Peak CK, IU/L	913 (321–2537)	861 (317–2390)	1420 (344–3148)	0.13
Peak CK-MB, IU/L	90 (24–252)	82 (24–245)	159 (24–301)	0.13
LVEF, %	55 (47–61)	55 (47–60)	55 (47–61)	0.93
Medication received at hospitalization				
Statin	128 (30.6)	100 (29.9)	28 (33.3)	0.55
ACE inhibitor/ARB	80 (19.1)	66 (19.8)	14 (16.7)	0.52
β-Blocker	27 (6.5)	23 (6.9)	4 (4.8)	0.48
Medication received at discharge				
Statin	394 (94.3)	313 (93.7)	81 (96.4)	0.34
ACE inhibitor/ARB	313 (74.9)	252 (75.4)	61 (72.6)	0.59
β-Blocker	281 (68.2)	222 (67.7)	59 (70.2)	0.65
Lesion location				0.82
LAD	230 (55.0)	184 (55.1)	46 (54.8)	
LCx	51 (12.2)	42 (12.6)	9 (10.7)	
RCA	137 (32.8)	108 (32.3)	29 (34.5)	
Multivessel disease	153 (36.6)	118 (35.3)	35 (41.7)	0.28
OCT characteristics				
Lesion length, mm	23.0 (18.0–28.8)	23.0 (16.0–28.0)	23.0 (18.0–32.6)	0.15
MLA, mm^2^	3.5 (2.4–4.6)	3.5 (2.4–4.6)	3.5 (2.3–4.7)	0.85
Minimum stent area, mm^2^	5.0 (4.0–6.4)	4.9 (4.0–6.5)	5.1 (4.1–6.3)	0.89
In-stent MLA, mm^2^	4.8 (3.9–6.1)	4.8 (3.9–6.3)	4.9 (4.1–5.8)	0.98
Average reference lumen area, mm^2^	6.3 (5.1–8.3)	6.3 (5.1–8.2)	6.2 (5.2–8.4)	0.86
Stent expansion ratio	0.77 (0.65–0.87)	0.77 (0.65–0.87)	0.76 (0.65–0.86)	0.55
NCL MLA, mm^2^	4.5 (3.0–6.5)	4.4 (3.0–6.6)	4.7 (3.0–6.3)	0.60
Irregular protrusion	213 (45.2)	175 (52.4)	38 (45.2)	0.24
Stent thrombus	116 (27.8)	95 (28.4)	21 (25.0)	0.53
Stent malapposition	290 (69.4)	234 (70.1)	56 (66.7)	0.55
Major stent edge dissection	8 (1.9)	7 (2.1)	1 (1.2)	0.59
TCFA in NCL	57 (13.7)	46 (13.8)	11 (13.1)	0.86

Values are median (IQR) or *n* (%).

ACE, angiotensin-converting enzyme; ARB, angiotensin II receptor blocker; BMI, body mass index; CABG, coronary artery bypass grafting; CK, creatine kinase; CKD, chronic kidney disease; CK-MB, creatine kinase–myocardial band; HbA1c, glycosylated haemoglobin; HDL-C, HDL cholesterol; LAD, left anterior descending artery; LCx, left circumflex artery; LDL-C, LDL cholesterol; LVEF, left ventricular ejection fraction; MI, myocardial infarction; MLA, minimum lumen area; NCL, non-culprit lesion; OCT, optical coherence tomography; PCI, percutaneous coronary intervention; RCA, right coronary artery; STEMI, ST-segment elevation myocardial infarction; TCFA, thin-cap fibroatheroma; TG, triglyceride.

### Outcomes

During a median follow-up of 961 days [interquartile range (IQR): 662–1355 days], 60 patients (14.4%) experienced TVF (TVF group), whereas 358 patients (85.6%) did not (non-TVF group; *Figure 2*). The TVF group included 17 (4.1%) patients who experienced cardiac death, 4 (1.0%) patients who experienced target vessel–related MI, and 44 (10.5%) patients who experienced ischaemia-driven TVR (*Table 2*). The 5-year cumulative incidence of TVF was 35.0%. The median follow-up period was 259 (IQR: 33–362) days in the TVF group [cardiac death: 37 (IQR: 10.5–63.5) days, target vessel–related MI: 252 (IQR: 7.5–620) days, and ischaemia-driven TVR: 304 (IQR: 195–434.5) days] and 1053 (IQR: 763–1424) days in the non-TVF group.

**Table 2:** Clinical outcomes

	Total (*n* = 418)
TVF, *n* (%)	60 (14.4)
Cardiac death, *n* (%)	17 (4.1)
Target vessel–related MI, *n* (%)	4 (1.0)
Ischaemia-driven TVR, *n* (%)	44 (10.5)
TLR, *n* (%)	22 (5.3)
Non-TLR TVF, *n* (%)	22 (5.3)

MI, myocardial infarction; TVF, target vessel failure; TVR, target vessel revascularization.

### Comparison between the target vessel failure and the non–target vessel failure groups

The TVF group had a significantly lower left ventricular ejection fraction (51 vs. 55%, *P* = 0.002), lower statin use frequency at discharge (83.3 vs. 96.1%, *P* < 0.001), and a higher incidence of multivessel disease (53.3 vs. 33.8%, *P* = 0.004) than those of the non-TVF group (see Supplementary material online, *Table S1*). The OCT characteristic values were significantly lower in the TVF group than those in the non-TVF group, as follows: minimum lumen area (2.5 vs. 3.6 mm^2^, *P* < 0.001), in-stent minimum lumen area (4.6 vs. 4.9 mm^2^, *P* = 0.04), average reference lumen area (5.7 vs. 6.4 mm^2^, *P* = 0.007), and minimum lumen area at NCL (2.9 vs. 4.8 mm^2^, *P* < 0.001). The TVF group had a significantly higher prevalence of irregular protrusion (75.0 vs. 46.9%, *P* < 0.001), stent thrombus (50.0 vs. 24.0%, *P* < 0.001), and TCFA in NCL (38.3 vs. 9.5%, *P* < 0.001) than the non-TVF group.

### Target vessel failure–associated factors in the conventional model

**Figure 3:**
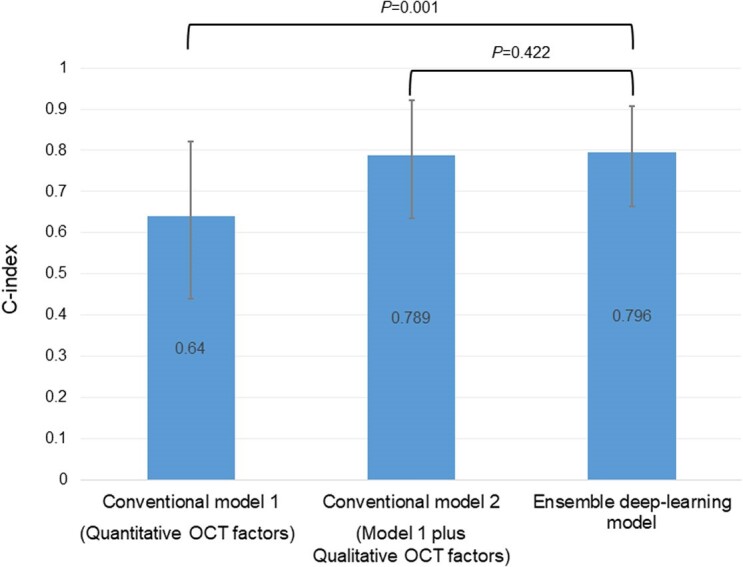
A comparison of model performance between conventional and deep-learning models. Harrell’s *C*-index values for conventional Model 1 (including only quantitative factors), conventional Model 2 (including quantitative and qualitative factors), and the ensemble deep-learning model are shown. The error bars represent a 95% confidence interval calculated using the bootstrap method.

The results of the multivariable Cox regression analysis for TVF are summarized in *Table 3*. The multivariable Model 1, which included only quantitative factors (minimum lumen area, minimum stent area, average reference lumen area, stent expansion ratio, and lesion length), showed that minimum lumen area [hazard ratio (HR): 0.57, 95% CI: 0.43–0.76, *P* < 0.001] was independently associated with TVF. The multivariable Model 2, which included quantitative and qualitative factors (Model 1 plus irregular protrusion, stent thrombus, stent malapposition, major stent edge dissection, and TCFA in NCL), showed that minimum lumen area (HR: 0.61, 95% CI: 0.46–0.82, *P* < 0.001), irregular protrusion (HR: 2.10, 95% CI: 1.09–4.05, *P* = 0.026), major stent edge dissection (HR: 4.38, 95% CI: 1.56–12.3, *P* = 0.005), and TCFA in NCL (HR: 3.89, 95% CI: 2.11–7.15, *P* < 0.001) were independently associated with TVF. The *C*-index values of Models 1 and 2 were 0.640 (95% CI: 0.440–0.822) and 0.789 (95% CI: 0.635–0.920), respectively (*Figure 3*). Additional Cox regression analyses including stent optimization criteria are described in Supplementary material online, *Table S2*.

**Table 3:** Cox regression analysis for factors associated with target vessel failure after percutaneous coronary intervention

	Model 1			Model 2		
	HR	95% CI	*P*-value	HR	95% CI	*P*-value
Quantitative factors						
MLA	0.57	0.43–0.76	<0.001	0.61	0.46–0.82	<0.001
Minimum stent area	1.17	0.72–1.89	0.53	1.07	0.67–1.73	0.77
Average reference lumen area	0.91	0.60–1.38	0.66	0.96	0.99–1.05	0.86
Stent expansion ratio	1.80	0.12–28.0	0.68	4.28	0.24–77.0	0.32
Lesion length	1.00	0.98–1.03	0.60	1.02	0.99–1.05	0.21
Qualitative factors						
Irregular protrusion				2.10	1.09–4.05	0.026
Stent thrombus				1.79	0.99–3.24	0.055
Stent malapposition				1.25	0.64–2.45	0.52
Major stent edge dissection				4.38	1.56–12.3	0.005
TCFA in NCL				3.89	2.11–7.15	<0.001

All variables are derived from post-PCI OCT findings.

CI, confidence interval; HR, hazard ratio; MLA, minimum lumen area; NCL, non-culprit lesion; PCI, percutaneous coronary intervention; TCFA, thin-cap fibroatheroma; TVF, target vessel failure.

### Patient-specific hazard and survival curves

**Figure 4:**
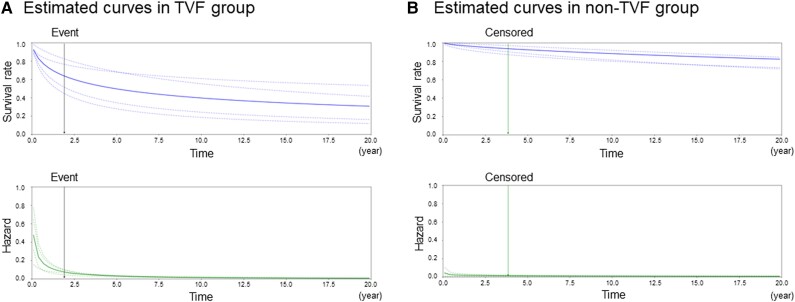
Representative hazard and survival curves in the target vessel failure and non-target vessel failure groups. Hazard and survival curves estimated using *µ* and *σ* outputs from the deep-learning model for individual patients are shown: (*A*) target vessel failure group and (*B*) non–target vessel failure group. The upper figure shows the survival curve, while the lower figure shows the hazard curve. The dotted lines represent the curves estimated from each cross-validation and the solid lines represent the curves resulting from the ensemble model averaged over each cross-validation. The black arrows indicate the time of the target vessel failure event and the green arrows indicate the time of censoring.

*Figure 4* illustrates the individualized cause-specific hazard and survival curves for the TVF (left graph) and non-TVF (right graph) groups. In the TVF group, the predicted hazard was relatively higher, especially in the early phase, leading to a correspondingly lower survival rate at earlier time points. Conversely, in the non-TVF group, the predicted hazard remained lower throughout, resulting in a consistently higher survival rate over time.

### Comparison of model performance between conventional and deep-learning models

The *C*-index values for the deep-learning models in the four-fold cross-validation were 0.696, 0.704, 0.661, and 0.679. The *C*-index for the ensemble deep-learning model was 0.796 (95% CI: 0.664–0.908; *Figure 3*). Compared with conventional Model 1, the deep-learning model showed a significantly higher predictive ability (*C*-index: 0.640 vs. 0.796, respectively, *P* = 0.001). Furthermore, the predictive ability of the deep-learning model was comparable to that of Model 2 (*C*-index: 0.789 vs. 0.796, respectively, *P* = 0.422; *Figure 3*).

### Visualization of the attention of deep-learning models

**Figure 5:**
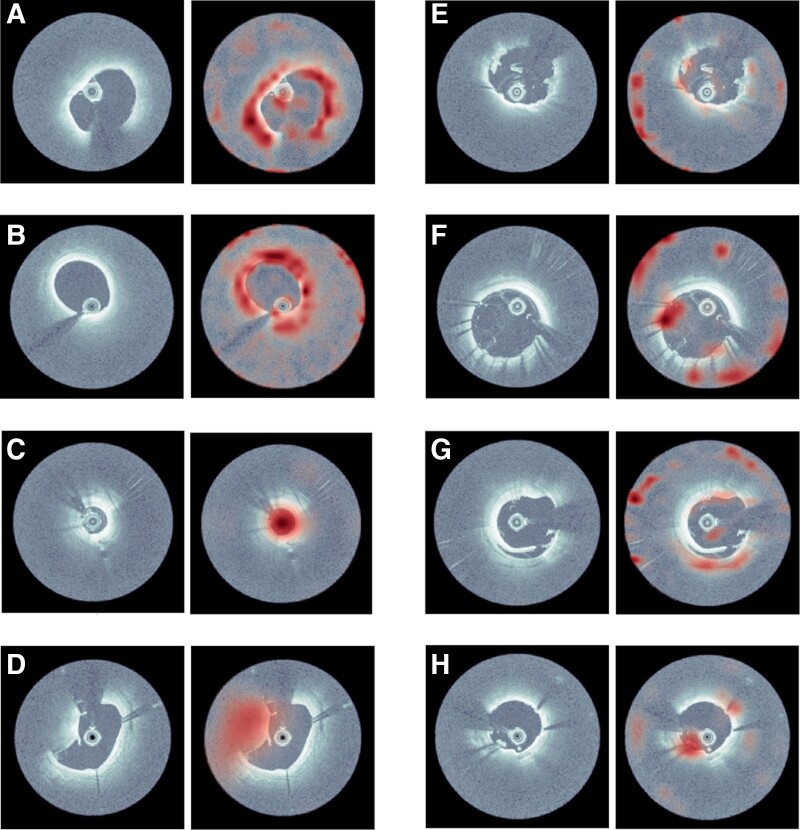
Visualization of the attention of deep-learning models. The original optical coherence tomography images (left panels) and heat map images highlighting the regions with the highest attention within each optical coherence tomography cross-sectional image (right panels). In (*A*) and (*B*), the highlighted areas correspond to thin-cap fibroatheroma and lipid-rich plaques. In (*C*), almost the entire lumen with a small area is highlighted. (*D*) and (*E*) may indicate calcified nodule and thrombus, respectively, whereas (*F*), (*G*), and (*H*) suggest stent malapposition, stent edge dissection, and irregular protrusion.

The model was visualized to enhance deep-learning model interpretation. As shown in the representative cases in *Figure 5*, GradCAM highlights the regions that cause greater activation of the network within the most ‘attended’ cross-sections determined by the attention mechanism. Most of the highlighted regions in the heat map correspond closely to the well-established characteristics observed by human observers. For instance, the highlighted areas in *A* and *B* correspond to thin-cap fibroatheroma and lipid-rich plaques, *C* highlights the vessel lumen with a small area, *D* and *E* potentially indicate calcified nodule and thrombus, and *F*, *G*, and *H* suggest stent malapposition, stent edge dissection, and irregular protrusion, respectively. The attention levels within each cross-section along the longitudinal axis are illustrated in Supplementary material online, *Figure S3*.

### Prospective validation

During a median follow-up period of 1084 days (IQR: 210–1491), 19 out of 89 (21.3%) patients experienced TVF events. A comparison of baseline characteristics and clinical events between the original and prospective cohorts is presented in Supplementary material online, *Table S3*. The *C*-index value of the prospective validation data set was 0.797 (95% CI: 0.669–0.908), which was comparable to that of the test data set in the original cohort.

## Discussion

To the best of our knowledge, this is the first study to develop a deep-learning survival model using intravascular OCT images. The main findings of this study can be summarized as follows: (i) using a CNN and transformer architecture, we successfully developed a deep-learning survival model that provides fully automated prognostic predictions based on post-PCI OCT images in patients with ACS; (ii) the deep-learning model significantly outperformed conventional models that included only quantitative factors and exhibited comparable predictive performance to the conventional model that incorporated both quantitative and qualitative factors, as assessed by expert analysts. This innovative model demonstrates the potential to overcome longstanding challenges in OCT analysis, such as time and effort requirements, as well as objectivity and reproducibility issues, potentially offering a significant advancement in the management of patients with ACS.

### Advantages of optical coherence tomography–based prognosis prediction model in patients with acute coronary syndrome undergoing percutaneous coronary intervention

Currently, ACS remains a challenging disease, with a significant number of patients experiencing secondary events.^18^ Given that a certain proportion of cardiovascular events are inevitable, risk stratification in the acute phase is essential. Secondary events in patients with ACS are known to stem from both stented culprit lesions and NCL. The PROSPECT study demonstrated that major adverse cardiovascular events were equally attributable to culprit lesions and NCL in patients with ACS who underwent PCI.^1^ Notably, intravascular imaging is useful in predicting future adverse events derived from both sources. Previous studies have consistently demonstrated that the post-PCI minimum stent area, stent edge dissection, and smaller reference lumen area evaluated using intravascular ultrasound or OCT are powerful predictors of future target lesion revascularization and stent thrombosis after PCI.^19^ In addition to the stented segment, we have previously demonstrated that OCT-based NCL findings, such as the presence of lipid-rich plaques and TCFA, were independently associated with subsequent TVF after PCI in patients with ACS.^5^ These findings highlight the clinical utility of OCT imaging for risk stratification of patients with ACS following PCI, not only at the lesion level, but also at a patient-wide level. However, despite the demonstrated benefits of intravascular imaging, its global adoption remains limited (e.g. <10% of cases in the USA between 2007 and 2017).^20^ Possible barriers include the additional time and cost associated with the procedures and the lack of adequate operator training for image interpretation.^21^ Therefore, to maximize the potential benefits of OCT in daily practice, it is imperative to develop user-friendly software with sufficient reliability. In this context, artificial intelligence technology could provide an innovative solution that would enable the widespread use of OCT during PCI.

Currently, many researchers are developing automated software to classify and segment atherosclerotic plaques using artificial intelligence, and several studies have validated its clinical availability.^22,23^ However, most previous deep-learning models were ‘classification models’, constructed using manual labelling provided by experienced readers. However, creating such teacher data requires significant time and effort, and the accuracy of this labelled data is often uncertain. In addition, detecting high-risk plaques cannot always predict future clinical events since not all vulnerable plaques lead to cardiovascular events. In contrast, our deep-learning model is unique in that it not only classifies atherosclerotic high-risk plaques but also directly predicts clinical outcomes after ACS. The teacher data in our deep-learning model consist solely of survival information, eliminating the need to create a large volume of labelled data. In practical terms, developing a robust survival model that predicts clinical outcomes based on vessel-level analysis proves more challenging than creating classification or segmentation models based on frame-level analysis using millions of cross-sectional OCT images. Nonetheless, the *C*-index for predicting TVF was 0.796, indicating a significantly better performance than that of conventional Model 1 (including only quantitative factors) and comparable to that of conventional Model 2 (including both quantitative and qualitative factors), highlighting its sufficient robustness for practical clinical application. Although this study could not demonstrate our model’s performance superiority over conventional Model 2, the time and effort saved compared with manual analysis was significant. In addition, deep-learning models can overcome the major problems of intra- and inter-observer reproducibility faced by manual OCT analysis.^24^

### Combination of convolutional neural network and transformer model

Many deep-learning models for medical image processing are based on CNN, which are adaptable to both two-dimensional and three-dimensional (3D) formats. Although it was possible to build a 3D-CNN model that directly inputs a single vessel, our approach chose to integrate the architecture of the transformer model. This model was originally developed for natural language processing and has been widely applied in various fields. In our model, each cross-sectional vessel image is linked to an individual ‘word’ in a sentence for natural language processing. A key advantage of the transformer is its ability to handle sequential data effectively and its capacity to easily utilize information from distant locations within an image, a task notably challenging for a model using only the CNN architecture. Another advantage of the transformer is its ability to visualize the most attended region using the ‘self-attention’ mechanism. *Figure 5* illustrates the regions with the highest attention related to future TVF events, most of which correspond to well-known OCT characteristics associated with adverse cardiovascular events, such as lipid-rich plaques, TCFA, thrombus, and small lumen area. Although not shown in the present study, the transformer model has the potential to reveal several novel OCT findings associated with future cardiovascular events. Alongside the combined architecture, we employed an ensemble model constructed by averaging the predictions of the top-performing models across different folds. These top models likely learned distinct aspects of the data, and the ensemble approach leverages this diversity, capturing a wider range of patterns. Consequently, this method reduces the risk of overfitting and mitigates individual model biases and errors, leading to more generalized and robust predictions.

### Comparison of deep-learning-based survival model and Cox proportional hazard model

Currently, the Cox proportional hazards model is the gold standard for determining prognostic variables. This semi-parametric model calculates the effects of observed variables on the risk of a prognostic event and assumes that the patient’s log risk of failure is a linear combination of the patient’s covariates.^25^ However, in many clinical settings, this assumption of linearity for the log-risk function may be overly simplistic. Hence, we developed a non-linear survival model using a novel artificial intelligence–based method that integrates a CNN and the transformer model. This non-linear survival model can predict an individual hazard function, assumed to follow a log-logistic distribution characterized by a location parameter *µ* and scale parameter *σ* for each patient. Additionally, the Cox proportional hazards model is limited in its ability to handle tabular data variables extracted by human observers, potentially overlooking image features that are still unknown but potentially relevant to prognosis. In contrast, our novel method circumvents these disadvantages as a fully automated survival model that requires only raw image data as input, underscoring its potential to revolutionize survival-prediction models for medical imaging.

### Limitations

This study had several limitations. First, the sample size for the patient-level survival model was small, and a larger sample size may provide greater predictive ability. Second, the imbalanced data set between the TVF and the non-TVF groups [i.e. the small number of patients in the TVF group (14.4%) and short median time to event in the TVF group (259 days)] may have reduced the predictive ability of this model. In the case of diseases such as cancer, which have a higher incidence of events and a longer observation period, this approach may provide a survival model with better performance. Third, external validation was lacking; instead, we opted for forward validation within the same participating facility. However, our model achieved a *C*-index of 0.797, demonstrating comparable performance to the internal test data set. This highlights the potential for future use in predicting post-procedural outcomes during PCI procedures.

## Conclusions

The CNN and transformer-based deep-learning model enabled fully automated prediction of cardiovascular outcomes after PCI in patients with ACS. The performance of the model was comparable to that of a conventional human-based model developed by expert clinicians. This deep-learning-based survival model holds promise for advancing the risk stratification of patients with ACS undergoing PCI.

## Lead author biography


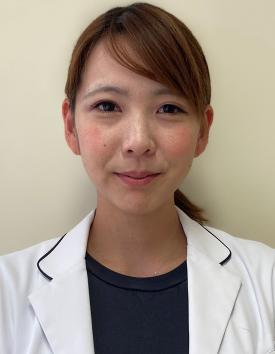
Tomoyo Hamana, MD, PhD, is currently a research fellow at CVPath Institute, USA, who previously worked at Kobe University. Her main academic interests include cardiovascular imaging and intervention, as well as histopathological and molecular biology research using multi-omics analysis.

## Supplementary Material

ztae067_Supplementary_Data

## Data Availability

The data underlying this article cannot be shared publicly for reasons of respecting the privacy of individuals who participated in the study. The data will be shared on reasonable request to the corresponding author.

## References

[1] Stone GW, Maehara A, Lansky AJ, De Bruyne B, Cristea E, Mintz GS, et al A prospective natural-history study of coronary atherosclerosis. N Engl J Med 2011;364:226–235. 21247313 10.1056/NEJMoa1002358

[2] Erlinge D, Maehara A, Ben-Yehuda O, Bøtker HE, Maeng M, Kjøller-Hansen L, et al Identification of vulnerable plaques and patients by intracoronary near-infrared spectroscopy and ultrasound (PROSPECT II): a prospective natural history study. Lancet 2021;397:985–995. 33714389 10.1016/S0140-6736(21)00249-X

[3] Patwari P, Weissman NJ, Boppart SA, Jesser C, Stamper D, Fujimoto JG, et al Assessment of coronary plaque with optical coherence tomography and high-frequency ultrasound. Am J Cardiol 2000;85:641–644. 11078281 10.1016/s0002-9149(99)00825-5

[4] Khan SU, Agarwal S, Arshad HB, Akbar UA, Mamas MA, Arora S, et al Intravascular imaging guided versus coronary angiography guided percutaneous coronary intervention: systematic review and meta-analysis. BMJ 2023;383:e077848. 37973170 10.1136/bmj-2023-077848 PMC10652093

[5] Kakizaki S, Otake H, Seike F, Kawamori H, Toba T, Nakano S, et al Optical coherence tomography fractional flow reserve and cardiovascular outcomes in patients with acute coronary syndrome. JACC Cardiovasc Interv 2022;15:2035–2048. 36182656 10.1016/j.jcin.2022.08.010

[6] Kuno T, Kiyohara Y, Maehara A, Ueyama HA, Kampaktsis PN, Takagi H, et al Comparison of intravascular imaging, functional, or angiographically guided coronary intervention. J Am Coll Cardiol 2023;82:2167–2176. 37995152 10.1016/j.jacc.2023.09.823

[7] Kang DY, Ahn JM, Yun SC, Hur SH, Cho YK, Lee CH, et al Optical coherence tomography-guided or intravascular ultrasound-guided percutaneous coronary intervention: the OCTIVUS randomized clinical trial. Circulation 2023;148:1195–1206. 37634092 10.1161/CIRCULATIONAHA.123.066429

[8] Shibutani H, Fujii K, Ueda D, Kawakami R, Imanaka T, Kawai K, et al Automated classification of coronary atherosclerotic plaque in optical frequency domain imaging based on deep learning. Atherosclerosis 2021;328:100–105. 34126504 10.1016/j.atherosclerosis.2021.06.003

[9] Holmberg O, Lenz T, Koch V, Alyagoob A, Utsch L, Rank A, et al Histopathology-based deep-learning predicts atherosclerotic lesions in intravascular imaging. Front Cardiovasc Med 2021;8:779807. 34970608 10.3389/fcvm.2021.779807 PMC8713728

[10] Qiu B, Huang Z, Liu X, Meng X, You Y, Liu G, et al Noise reduction in optical coherence tomography images using a deep neural network with perceptually-sensitive loss function. Biomed Opt Express 2020;11:817–830. 32133225 10.1364/BOE.379551 PMC7041484

[11] Cao S, Yao X, Koirala N, Brott B, Litovsky S, Ling Y, et al Super-resolution technology to simultaneously improve optical & digital resolution of optical coherence tomography via deep learning. Annu Int Conf IEEE Eng Med Biol Soc 2020;2020:1879–1882. 33018367 10.1109/EMBC44109.2020.9175777 PMC8116943

[12] Hong H, Jia H, Zeng M, Gutiérrez-Chico JL, Wang Y, Zeng X, et al Risk stratification in acute coronary syndrome by comprehensive morphofunctional assessment with optical coherence tomography. JACC Asia 2022;2:460–472. 36339358 10.1016/j.jacasi.2022.03.004 PMC9627809

[13] Kubo T, Shinke T, Okamura T, Hibi K, Nakazawa G, Morino Y, et al Optical frequency domain imaging vs. intravascular ultrasound in percutaneous coronary intervention (OPINION trial): one-year angiographic and clinical results. Eur Heart J 2017;38:3139–3147. 29121226 10.1093/eurheartj/ehx351 PMC5837511

[14] Li Z, Liu F, Yang W, Peng S, Zhou J. A survey of convolutional neural networks: analysis, applications, and prospects. IEEE Trans Neural Netw Learn Syst 2022;33:6999–7019. 34111009 10.1109/TNNLS.2021.3084827

[15] Vaswani A, Shazeer N, Parmar N, Uszkoreit J, Jones L, Gomez AN, et al Attention is all you need. Adv Neural Inf Process Syst 2017;30:3–5.

[16] Kaiming H, Xiangyu Z, Shaoqing R, Jian S. Deep residual learning for image recognition. In: *2016 IEEE Conference on Computer Vision and Pattern Recognition (CVPR)*, Las Vegas, NV, USA, 2016, p. 770–778.

[17] Popescu DM, Shade JK, Lai C, Aronis KN, Ouyang D, Moorthy MV, et al Arrhythmic sudden death survival prediction using deep learning analysis of scarring in the heart. Nat Cardiovasc Res 2022;1:334–343. 35464150 10.1038/s44161-022-00041-9 PMC9022904

[18] Alnasser SM, Huang W, Gore JM, Steg PG, Eagle KA, Anderson FA, et al Late consequences of acute coronary syndromes: global registry of acute coronary events (GRACE) follow-up. Am J Med 2015;128:766–775. 25554379 10.1016/j.amjmed.2014.12.007

[19] Prati F, Romagnoli E, Gatto L, La Manna A, Burzotta F, Limbruno U, et al Clinical impact of suboptimal stenting and residual intrastent plaque/thrombus protrusion in patients with acute coronary syndrome: the CLI-OPCI ACS substudy (Centro per la Lotta Contro L'Infarto-ptimization of percutaneous coronary intervention in acute coronary syndrome). Circ Cardiovasc Interv 2016;9:e003726. 27965297 10.1161/CIRCINTERVENTIONS.115.003726

[20] Fazel R, Yeh RW, Cohen DJ, Rao SV, Li S, Song Y, et al Intravascular imaging during percutaneous coronary intervention: temporal trends and clinical outcomes in the USA. Eur Heart J 2023;44:3845–3855. 37464975 10.1093/eurheartj/ehad430 PMC10567999

[21] Mintz GS . Intravascular imaging, stent implantation, and the elephant in the room. JACC Cardiovasc Interv 2017;10:2499–2501. 29153500 10.1016/j.jcin.2017.09.024

[22] Chu M, Jia H, Gutiérrez-Chico JL, Maehara A, Ali ZA, Zeng X, et al Artificial intelligence and optical coherence tomography for the automatic characterisation of human atherosclerotic plaques. EuroIntervention 2021;17:41–50. 33528359 10.4244/EIJ-D-20-01355 PMC9724931

[23] Lee J, Kim JN, Gharaibeh Y, Zimin VN, Dallan LAP, Pereira GTR, et al OCTOPUS—optical coherence tomography plaque and stent analysis software. Heliyon 2023;9:e13396. 36816277 10.1016/j.heliyon.2023.e13396 PMC9932655

[24] Brown AJ, Jaworski C, Corrigan JP, de Silva R, Bennett MR, Mahmoudi M, et al Optical coherence tomography imaging of coronary atherosclerosis is affected by intraobserver and interobserver variability. J Cardiovasc Med (Hagerstown) 2016;17:368–373. 26406395 10.2459/JCM.0000000000000304

[25] Katzman JL, Shaham U, Cloninger A, Bates J, Jiang T, Kluger Y. DeepSurv: personalized treatment recommender system using a Cox proportional hazards deep neural network. BMC Med Res Methodol 2018;18:24. 29482517 10.1186/s12874-018-0482-1 PMC5828433

