# Supplementary material for: Deep-learning-driven optical coherence tomography analysis for cardiovascular outcome prediction in patients with acute coronary syndrome
Source: Eur Heart J Digit Health. 2024 Sep 27;5(6):692–701. doi: 10.1093/ehjdh/ztae067 (PMC11570387; doi:10.1093/ehjdh/ztae067)
Supplement: ztae067_Supplementary_Data [file ztae067_supplementary_data.docx]

**SUPPLEMENTAL MATERIAL**

**Deep**-**Learning-Driven Optical Coherence Tomography Analysis for Cardiovascular Outcome Prediction in Patients with Acute Coronary Syndrome**

Author names:

Tomoyo Hamana*, Makoto Nishimori*, Satoki Shibata, Hiroyuki Kawamori, Takayoshi Toba, Takashi Hiromasa, Shunsuke Kakizaki, Satoru Sasaki, Hiroyuki Fujii, Yuto Osumi, Seigo Iwane, Tetsuya Yamamoto, Shota Naniwa, Yuki Sakamoto, Yuta Fukuishi, Koshi Matsuhama, Hiroshi Tsunamoto, Hiroya Okamoto, Kotaro Higuchi, Tatsuya Kitagawa, Masakazu Shinohara, Koji Kuroda, Masamichi Iwasaki, Amane Kozuki, Junya Shite, Tomofumi Takaya, Ken-ichi Hirata, Hiromasa Otake

*Tomoyo Hamana and Makoto Nishimori contributed equally to this work.

| **Table of contents** | **Page** |
| --- | --- |
| 1. **Participating institutions** 2. **Exclusion criteria** 3. **Definitions of clinical outcomes** 4. **OCT image analysis and definitions** 5. **Evaluation of the accuracy of conventional OCT analysis** 6. **Establishment of Cox regression models including stent optimization criteria** 7. **Data pre-processing** 8. **Prospective validation** 9. **Visualising decision basis** 10. **Reproducibility of conventional OCT analysis** 11. **Predictive ability of additional Cox regression models** 12. **Supplemental Table 1** 13. **Supplemental Table 2** 14. **Supplemental Table 3** 15. **Supplemental Figure 1** 16. **Supplemental Figure 2** 17. **Supplemental Figure 3** 18. **References** | **3**  **3**  **3–4**  **4–5**  **5–6**  **6**  **6–7**  **7**  **7–8**  **8**  **9**  **10–12**  **13**  **14–15**  **16**  **17**  **18**  **19–20** |

**Appendix**

**1. Participating institutions**

1) Kobe University Graduate School of Medicine, Kobe, Japan

2) Hyogo Prefectural Awaji Medical Centre, Sumoto, Japan

3) Osaka Saiseikai Nakatsu Hospital, Osaka, Japan

4) Hyogo Prefectural Himeji Cardiovascular Centre, Himeji, Japan

**2. Exclusion criteria**

The exclusion criteria were as follows: (i) patients with a left main trunk artery lesion, (ii) patients with a bypass graft lesion, (iii) patients without post-PCI OCT images, (iv) patients without examinable OCT images owing to poor image quality or final thrombolysis in myocardial infarction grade <2, and (v) patients without follow-up for at least 6 months.

**3. Definition of clinical outcomes**

Cardiac death, defined according to the Academic Research Consortium (ARC) definition,^1^ was a composite of any death due to a proximate cardiac cause (e.g., myocardial infarction, low-output failure, and fatal arrhythmia), unwitnessed death, death of unknown cause, and all procedure-related deaths, including those related to concomitant treatment. Target vessel-related myocardial infarction (MI) was defined as myocardial infarction in the vessel treated with the index PCI. Only spontaneous MI was included, while excluding procedural MI, based on the universal MI classification system.^2^ Specifically, diagnosis of spontaneous MI (Type 1, 2, 4b, and 4c) was characterised by clinical evidence consistent with acute myocardial ischaemia, whereas procedural MI (Type 4a) was characterised by increases of cardiac troponin (cTn) values within 48 hours post-PCI. In addition, MI with an indeterminant origin was not included in this endpoint. Ischaemia-driven target vessel revascularization (TVR) was defined as an unplanned repeat PCI or bypass graft placement for the vessel treated during the index PCI.

**4. OCT image analysis and definitions**

OCT was performed in patients with ACS after PCI, as previously reported.^3^ OCT images were acquired using a frequency-domain OCT system (ILUMIEN; Abbott Vascular, Santa Clara, CA, USA) with a Dragonfly™ Optis™ OCT imaging catheter (Abbott Vascular). Offline OCT analysis was performed using a dedicated software (Light Lab Imaging Inc., Westford, MA, USA). A 0.014-inch conventional standard guide wire was positioned distally in the target vessel, and the OCT catheter was advanced to the distal end of the target lesion. For image acquisition, blood in the lumen was replaced with contrast medium or low-molecular-weight dextran. OCT was performed at the end of the procedure beginning as far distal as possible to the ostium of the ACS culprit vessels using an integrated automated pullback device at 18 or 36 mm/s. The images were digitally stored offline. All OCT images were analysed by three experienced investigators (D.F., S.K., and Y.O.) who were blinded to the angiographic data and clinical presentations.

For quantitative analysis, the minimum lumen area, minimum stent area, in-stent minimum lumen area, average reference lumen area, and minimum lumen area at NCL were measured. Irregular protrusion was defined as the protrusion of material with an irregular surface into the lumen between the struts. As struts are occasionally buried within the intima, we only included in-stent protrusions with a maximal height of ≥100 μm for analysis. Stent thrombus was defined as a mass protruding into the lumen with significant attenuation behind the mass. Stent malapposition was defined as struts clearly separated from the vessel wall by ≥0.2 mm. Stent edge dissection was defined as disruption of the luminal surface with a visible flap at the stent edge or 5 mm proximal and distal reference segments. Major stent edge dissection was defined as a length of ≥60° of the circumference of the vessel and ≥3 mm in length. Thin-cap fibroatheroma (TCFA) was defined as a plaque with a fibrous-cap thickness of <65 μm and a lipid arc of ≥90°.

**5. Evaluation of the accuracy of conventional OCT analysis**

To assess the accuracy of conventional OCT analysis, we evaluated the intra- and inter-observer reproducibility between two independent observers (D.F. and S.K) in 30 randomly selected cases. For continuous variables, the intraclass correlation coefficient (ICC) assessing absolute agreement was used, and Bland-Altman plots were generated for paired observations to compare the mean against the difference in the measurements. For categorical analysis, observer variability was expressed as a Cohen’s kappa (*κ*) coefficient of agreement.^4^

**6. Establishment of Cox regression models including stent optimization criteria**

Two types of Cox regression models including the success or failure of optimal stent implantation and TCFA in NCL were established, based on different types of stent optimization criteria.^5-6^ Sub-optimal stent implantation was defined as meeting at least one of the criteria in the following stent optimization criteria:

1) CLI-OPCI II criteria: in-stent minimum lumen area <4.5 mm^2^, dissection thickness >200 µm at the distal stent edge, and distal or proximal reference lumen area <4.5 mm^2^.

2) FORZA criteria: major stent strut malapposition (>350 µm), major stent under expansion (in-stent minimal lumen area of <75% of the average reference lumen area), and major stent edge dissection (>600 µm in length).

**7. Data pre-processing**

To augment the diversity of the training data, 50 random slices from the entire coronary artery series were selected in each epoch as inputs to the learning model. Each input image was subjected to two types of random image processing, which varied with each epoch. First, random rotation involved randomly rotating the original image between 0° and 360°, with the same angle applied to all selected cross-sectional images. Secondly, random contrast control adjusted the pixel values of the original image using the formula: adjusted pixel value = [input pixel value] × alpha + beta, where alpha and beta were randomly determined from uniform distributions ranging from -1.5 to 1.5 and -0.3 to 0.3, respectively. Subsequently, values below 0 and above 1 were clipped to 0 and 1, respectively.

**8. Prospective validation**

Since external validation at other facilities proved difficult, prospective validation was performed within one of the participating facilities (Hyogo Prefectural Awaji Medical Centre) to further validate the model’s performance. We included 89 consecutive patients who underwent OCT-guided PCI for ACS between January 2019 and March 2021 for the prospective validation cohort. The inclusion and exclusion criteria were identical to those used for our original training, validation, and test cohorts.

**9. Visualising decision basis**

We implemented a two-step process to integrate the CNN and Transformer models for the visualisation of decision basis. Initially, we extracted the attention layers from the Transformer model and obtained 50 cross-sectional image slices from each vessel. Subsequently, we employed a Gradient-weighted Class Activation Mapping (GradCAM) technique^8^ to produce an activation map derived from the gradients of the final CNN layer in the Resnet50 model, which served as the embedding model for the cross-sectional images. The weights obtained in the initial step were applied to each image to generate a comprehensive heat map that elucidated the decision-making process.

**10. Reproducibility of conventional OCT analysis**

Intra-observer agreement was excellent for the quantitative analysis: average reference vessel area (ICC: 0.986, *P*<0.001), minimum stent area (ICC: 0.968, *P*<0.001), MLA (ICC: 0.996, *P*<0.001), in-stent MLA (ICC: 0.987, *P*<0.001), and lesion length (ICC: 1.000, *P*<0.001) (**Supplemental Figure 2**). Furthermore, intra-observer agreement was strong for the qualitative analysis: irregular protrusion (κ=0.842, 95% confidence interval [CI] 0.633–1.000), stent thrombosis (κ=0.798, 95%CI 0.582–1.000), stent malapposition (κ=0.737, 95%CI 0.505–0.968), stent edge dissection (κ=1.000, 95%CI 1.000–1.000), and TCFA in NCL (κ=0.706, 95%CI 0.396–1.000). Likewise, inter-observer agreement was excellent for the quantitative analysis: average reference vessel area (ICC: 0.974, *P*<0.001), minimum stent area (ICC: 0.946, *P*<0.001), MLA (ICC: 0.989, *P*<0.001), in-stent MLA (ICC: 0.984, *P*<0.001), and lesion length (ICC: 0.998, *P*<0.001) (**Supplemental Figure 2**). In contrast, inter-observer agreement for qualitative analysis was only acceptable; irregular protrusion (κ=0.791, 95%CI 0.571–1.000), stent thrombosis (κ=0.645, 95%CI 0.379–0.910), stent malapposition (κ=0.609, 95%CI 0.351–0.866), stent edge dissection (κ=0.651, 95%CI 0.021–1.000), and TCFA in NCL (κ=0.734, 95%CI 0.451–1.000).

**11. Predictive ability of additional Cox regression models**

The incidence of patients with sub-optimal stent implantation based on the CLI OPCI II and FORZA criteria was 51.7% and 67.9%, respectively, which was lower than those from the previous study.^8^ The results of the additional Cox regression analysis for factors associated with TVF after PCI are shown in **Supplemental Table 2**. The C-index values of those models were 0.44 (95%CI: 0.63–0.82) and 0.47 (95%CI: 0.66–0.84), respectively.

**Supplemental Table 1. Baseline and conventional human-based OCT characteristics between the TVF and non-TVF groups**

|  | **TVF group**  **(n=60)** | **Non-TVF group**  **(n=358)** | | ***P*-value** | |
| --- | --- | --- | --- | --- | --- |
| **Age, y** | 69 (63-80) | 68 (60-76) | | 0.21 | |
| **Male** | 44 (73.3) | 270 (75.4) | | 0.73 | |
| **BMI, kg/m^2^** | 23.6 (20.6-25.5) | 23.2 (21.3-25.3) | | 0.92 | |
| **Comorbidity** | | | | | |
| **Hypertension** | 41 (68.3) | 237 (66.2) | | 0.75 | |
| **Dyslipidaemia** | 34 (56.7) | 225 (62.8) | | 0.36 | |
| **Diabetes mellitus** | 23 (38.3) | 145 (40.5) | | 0.75 | |
| **Smoking** | 35 (58.3) | 220 (61.5) | | 0.65 | |
| **Family history** | 15 (25.0) | 59 (16.5) | | 0.11 | |
| **Haemodialysis** | 3 (5.0) | 7 (2.0) | | 0.15 | |
| **Prior MI** | 2 (3.3) | 15 (4.2) | | 0.76 | |
| **Prior PCI** | 4 (6.7) | 21 (5.9) | | 0.81 | |
| **Prior CABG** | 0 (0.0) | 2 (0.6) | | 0.56 | |
| **Clinical presentation** |  |  | | 0.29 | |
| **STEMI** | 36 (60.0) | 211 (58.9) | |  | |
| **Non-STEMI** | 20 (33.3) | 99 (27.7) | |  | |
| **Unstable angina** | 4 (6.7) | 48 (13.4) | |  | |
| **Laboratory data** | | | | | |
| **LDL-C, mg/dL** | 120 (98-134) | 126 (101-151) | | 0.16 | |
| **HDL-C, mg/dL** | 43 (38-53) | 46 (38-55) | | 0.55 | |
| **TG, mg/dL** | 103 (74-178) | 121 (78-194) | | 0.25 | |
| **HbA1c, %** | 6.0 (5.7-6.7) | 6.0 (5.6-6.7) | | 0.81 | |
| **Creatinine, mg/dL** | 0.81 (0.62-0.99) | 0.80 (0.69-0.93) | | 0.95 | |
| **Peak CK, IU/L** | 1300 (328-3720) | 900 (320-2402) | | 0.26 | |
| **Peak CK-MB, IU/L** | 135 (27-291) | 85 (22-249) | | 0.49 | |
| **LVEF, %** | 51 (41-58) | 55 (49-61) | | 0.002 | |
| **Medication received at hospitalization** | | | | | |
| **Statin** | 15 (25.0) | 113 (31.6) | | 0.31 | |
| **ACE inhibitor/ARB** | 10 (16.7) | 70 (19.6) | | 0.60 | |
| **β-blocker** | 5 (8.3) | 22 (6.1) | | 0.52 | |
| **Medication received at discharge** | | | | | |
| **Statin** | 50 (83.3) | 344 (96.1) | | <0.001 | |
| **ACE inhibitor/ARB** | 40 (66.7) | 273 (76.3) | | 0.11 | |
| **β-blocker** | 38 (70.4) | 243 (67.9) | | 0.71 | |
| **Lesion location** |  | |  | | 0.80 |
| **LAD** | 35 (58.3) | | 195 (54.5) | |  |
| **LCx** | 5 (8.3) | | 46 (12.8) | |  |
| **RCA** | 20 (33.3) | | 117 (32.7) | |  |
| **Multivessel disease** | 32 (53.3) | | 121 (33.8) | | 0.004 |
| **OCT characteristics** | | | | | |
| **Lesion length, mm** | 23.0 (18.0-33.0) | | 23.0 (16.0-28.2) | | 0.48 |
| **MLA, mm^2^** | 2.5 (1.6-3.4) | | 3.6 (2.6-4.8) | | <0.001 |
| **Minimum stent area, mm^2^** | 4.7 (3.6-6.1) | | 5.1 (4.1-6.5) | | 0.08 |
| **In-stent MLA, mm^2^** | 4.6 (3.2-5.8) | | 4.9 (4.0-6.3) | | 0.04 |
| **Average reference lumen area, mm^2^** | 5.7 (4.6-7.1) | | 6.4 (5.2-8.4) | | 0.007 |
| **Stent expansion ratio** | 0.80 (0.68-0.89) | | 0.77 (0.64-0.86) | | 0.27 |
| **NCL MLA, mm^2^** | 2.9 (1.7-5.4) | | 4.8 (3.3-6.7) | | <0.001 |
| **Irregular protrusion** | 45 (75.0) | | 168 (46.9) | | <0.001 |
| **Stent thrombus** | 30 (50.0) | | 86 (24.0) | | <0.001 |
| **Stent malapposition** | 45 (75.0) | | 245 (68.4) | | 0.31 |
| **Major stent edge dissection** | 5 (8.3) | | 3 (0.8) | | <0.001 |
| **TCFA in NCL** | 23 (38.3) | | 34 (9.5) | | <0.001 |

Values are median (IQR) or n (%).

ACE=angiotensin-converting enzyme, ARB=angiotensin II receptor blocker, BMI=body mass index, CABG=coronary artery bypass grafting, CK=creatine kinase, CKD=chronic kidney disease, CK-MB=creatine kinase-myocardial band, HbA1c=glycosylated haemoglobin, HDL-C=high-density lipoprotein cholesterol, LAD=left anterior descending artery, LCx=left circumflex artery, LDL-C=low-density lipoprotein cholesterol, LVEF=left ventricular ejection fraction, MI=myocardial infarction, MLA=minimum lumen area, NCL=non-culprit lesion, OCT=optical coherence tomography, PCI=percutaneous coronary intervention, RCA=right coronary artery, STEMI=ST-segment elevation myocardial infarction, TCFA=thin-cap fibroatheroma, TG=triglyceride, TVF=target vessel failure.

**Supplemental Table 2. Additional Cox regression analysis for factors associated with TVF after PCI**

|  | **Model 3** | | | **Model 4** | | |
| --- | --- | --- | --- | --- | --- | --- |
|  | **HR** | **95% CI** | ***P*-value** | **HR** | **95% CI** | ***P*-value** |
| **Optimal stent implantation (CLI OPCI II criteria)** | 0.64 | 0.36-1.14 | 0.13 | ― | ― | ― |
| **Optimal stent implantation (FORZA criteria)** | ― | ― | ― | 1.63 | 0.92-2.90 | 0.093 |
| **TCFA in NCL** | 4.30 | 2.39-7.72 | <0.001 | 4.14 | 2.30-7.43 | <0.001 |

CI=confidence interval, HR=hazard ratio, NCL=non-culprit lesion, PCI=percutaneous coronary intervention, TCFA=thin-cap fibroatheroma, TVF=target vessel failure.

**Supplemental Table 3. Comparison between the original and prospective validation datasets**

|  | **Original dataset (n=418)** | **Prospective validation dataset (n=89)** | ***P*-value** |
| --- | --- | --- | --- |
| **Baseline characteristics** |  |  |  |
| **Age, y** | 68 (60-76) | 71 (62-78) | 0.17 |
| **Male** | 314 (75.1) | 69 (77.5) | 0.69 |
| **Comorbidities** |  |  |  |
| **Hypertension** | 278 (66.5) | 52 (58.4) | 0.18 |
| **Dyslipidaemia** | 259 (62.0) | 53 (59.6) | 0.72 |
| **Diabetes mellitus** | 168 (40.2) | 34 (38.6) | 0.81 |
| **Haemodialysis** | 10 (2.4) | 2 (2.2) | 1.00 |
| **Prior MI** | 17 (4.1) | 6 (6.7) | 0.27 |
| **Prior PCI** | 25 (6.0) | 12 (13.5) | 0.022 |
| **Prior CABG** | 2 (0.5) | 1 (1.1) | 0.44 |
| **Clinical presentation** |  |  | 0.38 |
| **STEMI** | 247 (59.1) | 57 (64.0) |  |
| **Non-STEMI** | 119 (28.5) | 19 (21.3) |  |
| **Unstable angina** | 52 (12.4) | 13 (14.6) |  |
| **Lesion location** |  |  | 0.28 |
| **LAD** | 230 (55.0) | 42 (47.2) |  |
| **LCx** | 51 (12.2) | 10 (11.2) |  |
| **RCA** | 137 (32.8) | 37 (41.6) |  |
| **Clinical outcomes** |  |  |  |
| **TVF** | 60 (14.4) | 19 (21.3) | 0.11 |
| **Cardiac death** | 17 (4.1) | 7 (7.9) | 0.16 |
| **Target vessel-related MI** | 4 (1.0) | 0 (0.0) | 1.00 |
| **Ischaemia-driven TVR** | 44 (10.5) | 12 (13.5) | 0.46 |
| **TLR** | 22 (5.3) | 8 (9.0) | 0.21 |
| **Non-TLR TVR** | 22 (5.3) | 4 (4.5) | 1.00 |
| **Duration period, days** | 960 (662-1355) | 1084 (210-1591) | 0.66 |

Values are median (IQR) or n (%).

CABG=coronary artery bypass grafting, LAD=left anterior descending artery, LCx=left circumflex artery, MI=myocardial infarction, PCI=percutaneous coronary intervention, RCA=right coronary artery, STEMI=ST-segment elevation myocardial infarction, TLR=target lesion revascularization, TVF=target vessel failure, TVR=target vessel revascularization.

**Supplemental Figure 1. Subsegments analysed using optical coherence tomography**


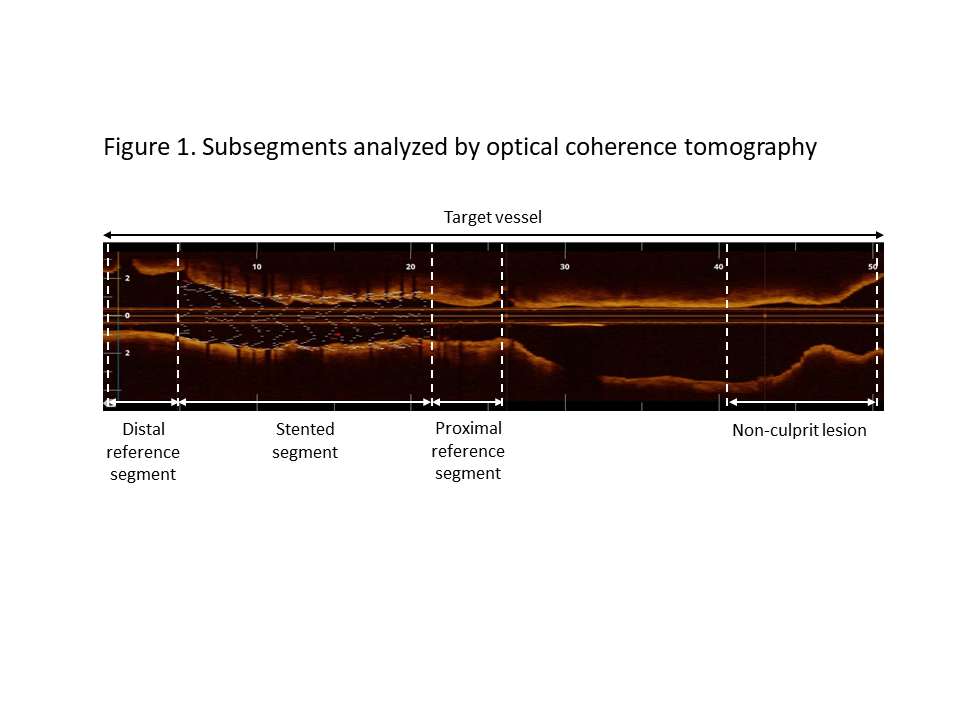


The target vessel was divided into the following longitudinal subsegments: 1) stented segment; 2) adjacent reference segments (5 mm in length); and 3) non-culprit lesion (NCL). A NCL was defined as an untreated coronary segment with >30% diameter stenosis on angiography and at least 5 mm away from the stent. If multiple candidate NCLs were present, the most stenotic lesion was defined as the NCL for that case.

**Supplemental Figure 2. Reproducibility of conventional OCT analysis**

**
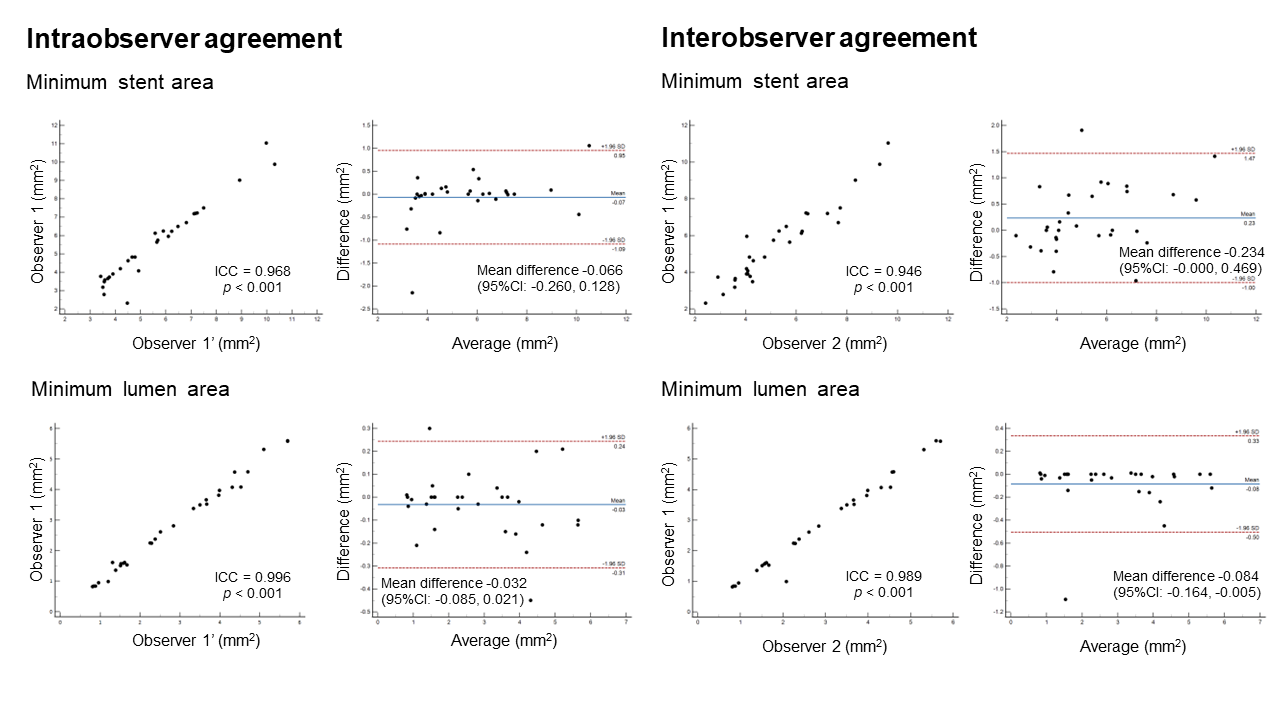
**

Intra-observer agreement of Observer 1 (left) and interobserver agreement between two independent experienced observers, Observers 1 and 2 (right). Scatter plot and Bland-Altman plots for minimum stent area (upper panels) and minimum lumen area (lower panels) illustrating correlations between observers.

ICC=intraclass correlation coefficient, CI=confidence interval.

**Supplemental Figure 3. Longitudinal data highlighted by ‘attention’ mechanism**


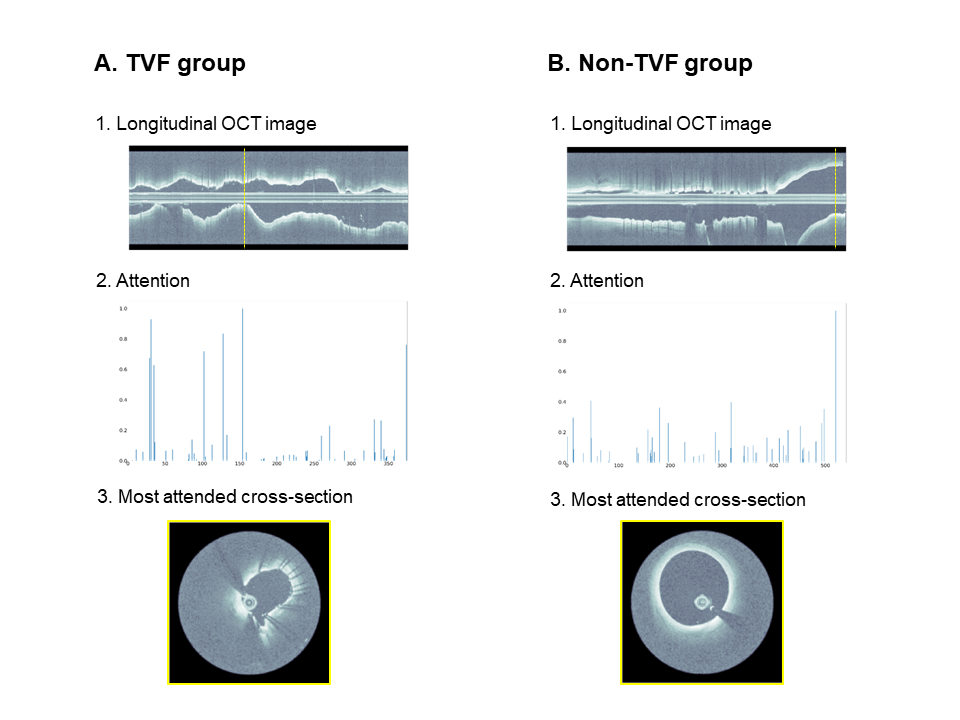


Representative TVF ( A) and non-TVF (B) cases in the left and right columns, respectively. The upper panels (A1 and B1) display longitudinal OCT images. The middle panels (A2 and B2) demonstrate the attention levels of 50 randomly selected cross-sections. In A2, the blue bows indicate the attention levels for predicting TVF; however, in B2, they indicate the levels for predicting non-TVF. The lower panels (A3 and B3) present the cross-sectional images that attracted the most attention in each case.

**References**

1. Cutlip DE, Windecker S, Mehran R, Boam A, Cohen DJ, van Es GA et al. Clinical end points in coronary stent trials: a case for standardized definitions. Circulation. 2007;115:2344-51.
2. Thygesen K, Alpert JS, Jaffe AS, Chaitman BR, Bax JJ, Morrow DA, et al. Fourth Universal Definition of Myocardial Infarction (2018). J Am Coll Cardiol. 2018;72:2231-64. doi: 10.1016/j.jacc.2018.08.1038.
3. Otake H, Kubo T, Takahashi H, et al. Optical Frequency Domain Imaging Versus Intravascular Ultrasound in Percutaneous Coronary Intervention (OPINION Trial): Results From the OPINION Imaging Study. JACC Cardiovasc Imaging 2018;11:111-23. doi: 10.1016/j.jcmg.2017.06.021
4. Brown AJ, Jaworski C, Corrigan JP, de Silva R, Bennett MR, Mahmoudi M, et al. Optical coherence tomography imaging of coronary atherosclerosis is affected by intraobserver and interobserver variability. J Cardiovasc Med (Hagerstown). 2016;17:368-73.
5. Prati F, Romagnoli E, Burzotta F, Limbruno U, Gatto L, La Manna A, et al. Clinical Impact of OCT Findings During PCI: The CLI-OPCI II Study. JACC Cardiovasc Imaging. 2015;8:1297-1305. doi: 10.1016/j.jcmg.2015.08.013.
6. Burzotta F, Leone AM, Aurigemma C, Zambrano A, Zimbardo G, Arioti M, et al. Fractional Flow Reserve or Optical Coherence Tomography to Guide Management of Angiographically Intermediate Coronary Stenosis: A Single-Center Trial. JACC Cardiovasc Interv. 2020;13:49-58. doi: 10.1016/j.jcin.2019.09.034.

7. Selvaraju RR, Cogswell M, Das A, Vedantam R, Parikh D, Batra D. Grad-cam: visual explanations from deep networks via gradient-based localization. In: *Proceedings of the IEEE international conference on computer vision.* 2017 (pp. 618-26).

8. Romagnoli E, Burzotta F, Vergallo R, Gatto L, Biondi-Zoccai G, Ramazzotti V, et al. Clinical impact of OCT-derived suboptimal stent implantation parameters and definitions. Eur Heart J Cardiovasc Imaging. 2023;25:48-57. doi: 10.1093/ehjci/jead172.
